# An analysis of the views of different members of the inpatient team on the role of the physician associate on the general adult psychiatric wards

**DOI:** 10.1192/bjo.2021.399

**Published:** 2021-06-18

**Authors:** Declan Hyland, Mohammed Uddin

**Affiliations:** 1 Consultant Psychiatrist, Clock View Hospital, Liverpool, Mersey Care NHS Foundation Trust; 2 Physician Associate, Clock View Hospital, Liverpool, Mersey Care NHS Foundation Trust

## Abstract

**Aims:**

Physician Associates (PAs) are healthcare professionals who have a general medical education background, having completed a two-year postgraduate degree. Whilst the number of PAs employed in healthcare trusts continues to increase, the number working in mental health settings remains small.

Mersey Care NHS Foundation Trust employed two PAs two years ago. In August 2019, a third PA was recruited to work at Clock View Hospital, a general adult inpatient unit.

This analysis aimed to establish the views of different members of the team across the three general adult wards and the Psychiatric Care Unit (PICU) at Clock View Hospital on the role of the PA.

**Method:**

A sample of members of staff was identified from across the three general adult inpatient wards at and the PICU, comprising: senior doctors (Consultants and Specialty Doctor), junior trainees (Core Trainee and Foundation Trainees), Ward Manager, Deputy Ward Manager, Band 5 nurse and Assistant Practitioner. Each member of staff was asked to answer the question “On a scale of 1 to 10 (with “1” being completely unhappy, “10” being completely happy), how happy are you to have a PA working on your ward?” Each staff member was then asked to provide comments on their views on the role of the PA.

**Result:**

Twenty-three members of staff participated – 3 x senior doctors, 4 x junior trainees, 4 Ward Managers, 4 Deputy Ward Managers, 4 x Band 5 nurses and 4 x Assistant Practitioners. The respondents were distributed equally across the three general adult wards and the PICU. All 23 members of staff provided a score of 10 out 10 to the question about how happy they were to have a PA working on the ward. Many of the staff members provided some very positive comments on their respective views about the role of the PA at Clock View Hospital. No negative comments were provided by any members of staff.

**Conclusion:**

It is clear from the large sample of members of staff of different grade at Clock View Hospital that were surveyed that the PA has been a warmly received and welcome addition to the inpatient team and that the PA is viewed as having become an important and valued member of the inpatient team. This provides a strong argument for both Mersey Care NHS Foundation Trust, and other mental health trusts across the U.K., to consider employing more PAs to work in their inpatient units.

